# UWB-gestures, a public dataset of dynamic hand gestures acquired using impulse radar sensors

**DOI:** 10.1038/s41597-021-00876-0

**Published:** 2021-04-12

**Authors:** Shahzad Ahmed, Dingyang Wang, Junyoung Park, Sung Ho Cho

**Affiliations:** Department of Electronic Engineering, Hanyang University, Seoul, South Korea

**Keywords:** Scientific community, Electrical and electronic engineering

## Abstract

In the past few decades, deep learning algorithms have become more prevalent for signal detection and classification. To design machine learning algorithms, however, an adequate dataset is required. Motivated by the existence of several open-source camera-based hand gesture datasets, this descriptor presents UWB-Gestures, the first public dataset of twelve dynamic hand gestures acquired with ultra-wideband (UWB) impulse radars. The dataset contains a total of 9,600 samples gathered from eight different human volunteers. UWB-Gestures eliminates the need to employ UWB radar hardware to train and test the algorithm. Additionally, the dataset can provide a competitive environment for the research community to compare the accuracy of different hand gesture recognition (HGR) algorithms, enabling the provision of reproducible research results in the field of HGR through UWB radars. Three radars were placed at three different locations to acquire the data, and the respective data were saved independently for flexibility.

## Abstract (summary)

**Table:** 

Measurement(s)	hand gesture • voluntary movement behavior
Technology Type(s)	ultra-wideband impulse radar • Imaging Technology
Factor Type(s)	type of hand gesture • participant
Sample Characteristic - Organism	Homo sapiens
Sample Characteristic - Environment	human house

Machine-accessible metadata file describing the reported data: 10.6084/m9.figshare.13181240

## Background & Summary

Hand gesture recognition (HGR) provides a convenient and natural method of human-computer interaction. User-friendly interfaces for human-machine interactions can be built using hand gestures. In HGR, gesture data are first acquired using a suitable sensor, and then patterns within the acquired sensory signals are recognized to identify different hand movements. Several sensors exist for data acquisition, including wearable devices^1^, cameras^2^, and radar sensors. Recently, radar has been considered a key enabling technology for HGR due to its many benefits over other sensors; for example, radar is less prone to lightning conditions than camera-based sensors. In addition, radar-based HGR systems do not require wearable devices. Currently, several commercial devices (such as Google Pixel 4 smartphones) are equipped with built-in radar for HGR.

Deep learning algorithms have shown great potential for the recognition and classification of hand gestures. Recent studies demonstrated a high classification accuracy for ultra-wideband (UWB) radar-based hand gesture classification^3,4^, where several sliding and circular hand motions were classified using deep learning approaches. Similarly, researchers from Google designed and manufactured a miniature radar named Soli solely for hand gesture recognition and sensing using a random forest classifier driven by low-dimensional features^5^. Recently, Wang *et al*.^6^ used the same Soli radar sensor^5^ to classify eleven different gestures using a convolutional neural network (CNN)-based classifier. Another study^7^ recognized six different gestures with radar sensors intended for vehicular and infotainment interfaces, and the classification output (class) was fed to an Android system to perform the desired operation based on gestures. Furthermore, a system called Radar Categorization for Input & Interaction (RadarCat)^8^ was established to provide a set of applications, including gesture recognition-based random forest classifiers. Recently, Park *et al*.^9^ focused on providing both radar hardware and a recognition algorithm for hand gestures; six different gestures were classified using long short-term memory (LSTM). The aforementioned studies suggest that machine learning-based solutions will enable radar sensors to have considerable positive impacts on HGR. However, machine learning algorithms are based on learning paradigms and therefore require some overhead, such as sufficient computing power and the need for a sufficiently large dataset to train the algorithm. Without an adequate hardware assembly and acquisition environment, it is often not possible to develop and test deep learning frameworks. Thus, to build HGR algorithms without purchasing hardware, a public dataset of hand gestures acquired through radar sensors is needed.

Several (signal and image) datasets for training deep learning algorithms are available to the public for download. For instance, ImageNet^10^ and LabelMe^11^ provide large collections of images intended for use in visual object recognition. These datasets eliminate the need to acquire images to test different machine learning algorithms and simultaneously provide a competitive platform for comparing the performance of different algorithms in similar environments. Recently, a small collection of governmental response events for COVID-19^12^ was released, and over 13,000 policy announcements were made by the governments of 195 countries; this public dataset can be used to train a CNN for the detection of COVID-19. Similarly, PhysioBank^13^ presents a collection of over 75 datasets containing samples of different biomedical signals, such as cardiopulmonary and neural signals, from both patients and healthy individuals. However, few vision-based hand-gesture datasets exist; among them are the Cambridge Hand Gesture Database (released in 2009) containing nine hundred sequences of images for nine different hand gesture classes^14^, MSRGesture3D (released in 2012)^15^ and EgoGesture^16^ (released in 2018). Furthermore, the RGBD-HuDaAct^17^ dataset provides a human activity recognition dataset acquired with a video camera and a depth sensor. Pisharady and Serbeck^18^ reported a comprehensive review of all available vision-based hand gesture datasets, and recently, a dataset of continuous-wave radar datasets for vital signs and heartbeats with six different human subjects recorded over 223 minutes was released^19^. However, no such public radar dataset exists for hand gestures. For all the studies regarding HGR with radar and other radio sensors, researchers first collect training data before testing their algorithms.

**Fig. 1:**
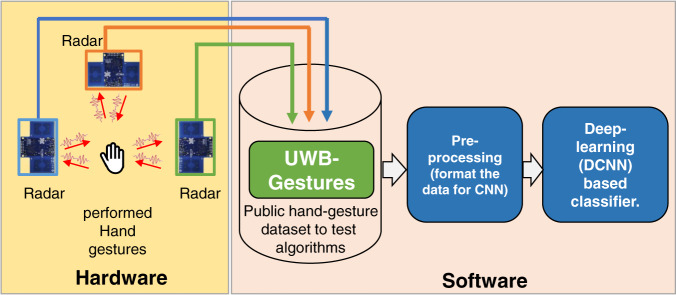
Summary of the overall workflow: collection of the UWB-Gestures dataset using three UWB radars and an application example of classification with a DCNN.

In this paper, we present the first-ever dataset of hand gestures collected using ultra-wideband (UWB) impulse radar. We expect that this dataset may eliminate the need to acquire data for algorithm testing and will provide a competitive environment for the research community to compare the accuracy of existing and newly proposed algorithms. The overall summary and the scope of this study are presented in Fig. 1. Three different radar sensors operating independently in a monostatic configuration are deployed, and the data from each radar sensor are saved separately in the repository. Consequently, the evaluation of HGR algorithms can be performed either by using a single radar sensor or by exploiting signals from multiple radar sensors simultaneously. An application example of a CNN-based classifier, as explained in Fig. 1, is also demonstrated in a subsequent section.

## Methods

### Literature survey-based selection of hand gestures

First, we performed a brief literature review to select hand gestures since there is no existing standard for selecting radar sensor-collected hand gestures to test HGR algorithms. Researchers always select a gesture set randomly to evaluate their algorithms. However, studies suggest that the nature of gestures is usually the same, i.e., swiping, sliding, pushing and cyclic rotation, among other movements. For example, Kim and Tomajian^20^ used 10 gestures of a similar nature to perform HGR using Doppler radar. Khan *et al*.^21^ used UWB radar to classify five gestures comprising hand sliding and pointing gestures acquired from three different human volunteers. Similarly, Ryu *et al*.^22^ constructed a feature-based gesture recognition algorithm and tested it on 7 hand gestures, including moving the hand left, right, up, down, clockwise, and counterclockwise and pushing the hand. Recent studies on radar sensor-based HGR^20–26^ used gestures of a similar nature to test their algorithms. Nevertheless, although the gestures were similar in nature, the data acquisition environment and type of radar differed among each study. As a result, the performance evaluation of each new algorithm varies as a function of the recorded dataset. Moreover, in all of the aforementioned studies, the datasets used for training and evaluating the algorithms consisted of a small number of samples. For example, the dataset used in Fhager *et al*.^26^ comprises only 180 samples per gesture. Ryu *et al*.^22^ performed each of their 7 gestures only 15 times for training purposes. A small number of training samples and human participants may cause a machine learning algorithm to be biased and lead to overfitting given only the known data samples; as a result, the algorithm may not be robust enough against unknown test data samples. To cope with the aforementioned challenges, the presented dataset has the following features:

- UWB-Gestures contains most (if not all) of the previously used gestures in radar-based HGR studies, as there is no procedural standard for acquiring hand gestures.
- Multiple volunteers were used to acquire the data to provide larger intragesture variations.
- Multiple (three) radar sensors were used for data acquisition, and the data of each radar sensor are separately accessible to provide flexibility in terms of preferences for hardware placement and number of radar sensors. Multiple radar sensors also permit hand tracking and trajectory prediction.

**Fig. 2:**
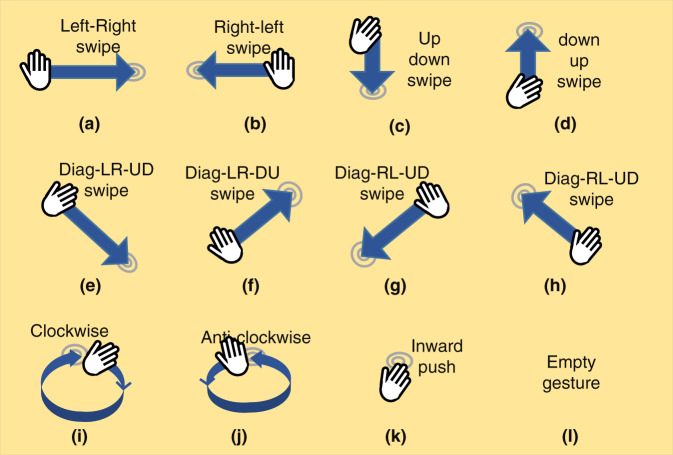
Gesture vocabulary: (**a–l**) show the twelve selected hand gestures for the UWB-Gestures dataset.

In this paper, we present the first-ever public dataset (called UWB-Gestures) of twelve different dynamic hand gestures, as presented in Fig. 2(a–l), where the gestures were acquired with UWB impulse radar. The modality of the video of each of the performed gestures is available at (http://casp.hanyang.ac.kr/uwbgestures). We selected eight swiping (sliding) gestures, as shown in Fig. 1(a–h): left-right (LR) swipe, right-left (RL) swipe, up-down (UD) swipe, down-up (DU) swipe, diagonal (diag)-LR-UD swipe, diag-LR-DU swipe, diag-RL-UD swipe and diag-RL-DU swipe. Additionally, Fig. 2(i–k) present clockwise rotation (CW), counterclockwise (CCW) rotation, and an inward push gesture. One empty gesture was added for each user to permit gesture and nongesture classification. The hands shown in Fig. 2 represent the starting point of each gesture. As stated earlier, these gestures were carefully selected based on the preferences of other researchers.

### Participants

The data were gathered from eight different participants to introduce more intragesture variations. Table 1 presents the details of each individual participant that can be used for analyzing intragesture and intergesture variations with respect to different hand sizes. The average age of the participants was 25.75 years old, and the average body mass index (BMI) was 22.19 ± 5 kg/m^2^. Although most of the human participants involved in the data acquisition process were from research occupations, they were provided with basic training prior to data acquisition.

**Table 1:** Details of the included human volunteers.

Volunteer	Age (years)	Weight (kg)	BMI
1	28	67	24
2	26	88	27.17
3	29	56	18.20
4	24	65	19.9
5	21	57	19.70
6	23	65	21.75
7	24	75	21.9
8	31	82	25
**Average**	**25.75**	**69**	**22.19**

### Data acquisition hardware

**Fig. 3:**
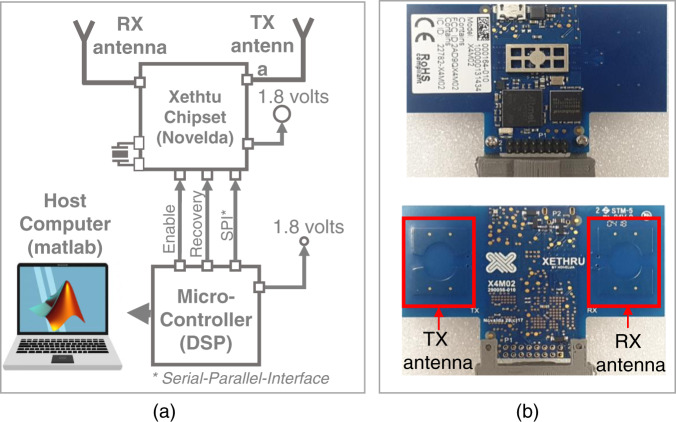
(**a**) Connectivity diagram: (**a**) UWB radar sensor and (**b**) front and back of the UWB radar sensor.

For data acquisition, we selected the XeThru X4 UWB impulse radar sensor from Novelda (Norway) due to its small size and extensive usage in different radar sensor-based applications, such as gesture recognition^3,21,25^, vital sign monitoring^27,28^ and automobiles^29^. The detailed technical specifications of the radar sensor are listed in Table 2. As shown in Table 2, the Novelda radar sensor is a UWB impulse radar sensor with a bandwidth of 2 GHz centered at a frequency of 8.745 GHz. The connectivity diagram is shown in Fig. 3(a), which demonstrates that the Novelda radar sensor consists of a pair of transmitter (Tx) and receiver (Rx) antennas and a DSP microcontroller that is further connected to a host computer, where MATLAB is used to collect and save the data. The front and back of the complete radar chip are shown in Fig. 3(b). The dataset was collected at Hanyang University, Seoul, South Korea.

**Table 2:** Technical specifications of the used radar.

Parameter	Specification
Accuracy	~1 millimeter
Center frequency	8.745 GHz
Sampling frequency	23 GHz
Frame rate	20 frames/second
Bandwidth (–10 dB)	1.5 GHz
Pulse repetition frequency	40.5 MHz
Antenna beamwidth	65 degrees
Number of antennas	1 pair of Tx and Rx antennas

Unlike traditional narrowband radar sensors, UWB radar transmits a signal with a wide frequency spectrum for a very short duration. For every sequence of transmitted impulse-like signals, the corresponding received signal x[n] consists of the reflected signal from N different paths and an additive noise term^30^. As a result, the received UWB signal is a linear combination of these N delayed and distorted signals and can be represented by:

1 $$x[n,k]=\mathop{\sum }\limits_{i=1}^{{N}_{path}}{a}_{ni}s\left(n,k-{T}_{i}\right)+N$$

where s[n] represents the estimate of the transmitted pulse shape received at the receiver that is usually distorted due to several different factors, such as the reflection, refraction and scattering coefficients of objects, and N represents additive noise. In addition, a_*ni*_ and T_i_ represent the scaling factor and duration, respectively, of the signal.

The terms N and K in Eq. (1) represent the rows and columns of the 2D received radar signal matrix, known as the fast time and slow time, respectively^30^. Here, the fast time (rows) of the radar signal matrix expresses the distance of the hand from the radar, while the slow time (columns) represents the frames transmitted by the radar (the duration of the hand gesture). The signal represented in Eq. (1) contains both the reflections from the target (hand) and the unwanted reflections from static objects within the operational area of the radar sensor, such as the human body. These unwanted reflections are usually termed clutter. The final 2D matrix containing the received hand reflections for one single gesture movement against an individual radar signal can be expressed as:

2 $${X}_{90,189}=\left[\begin{array}{ccc}{x}_{90,1} & \cdots \, & {x}_{90,198}\\ \vdots  & \ddots  & \vdots \\ {x}_{1,1} & \cdots \, & {x}_{1,189}\end{array}\right]$$

In this paper, hand gestures were recorded for 4.5 seconds, which corresponds to 90 (slow-time) rows. We adjusted the range of radar to 1.2 meters, yielding 189 fast-time samples.

### Data acquisition setup

**Fig. 4:**
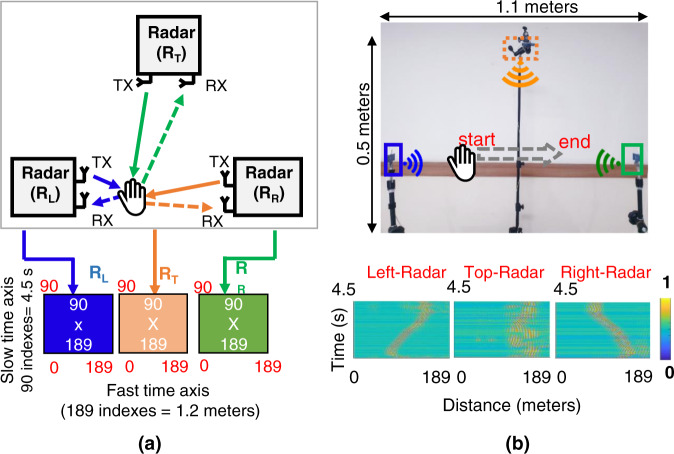
(**a**) Conceptual diagram of the hardware environment consisting of three radar sensors operating independently in a monostatic configuration; (**b**) demonstration of gesture 1 (LR swipe) in an actual experimental environment along with the signal patterns of all three radars.

The conceptual acquisition setup is presented in Fig. 4(a), which comprises 3 radars named R_L_, R_T_ and R_R_ (placed at the left, top and right sides, respectively, of the experimental setup). All three radars operate independently in a monostatic configuration, and signal transmission and reception are performed independently by each radar. The gestures were performed in the middle of all three radars. The distance between the left and right radars was 1.1 meters, and the distance between the midpoint of the horizontal radars and the top radar was 0.55 meters. Figure 4(b) illustrates the actual experimental setup and the data matrices against all three radars for gesture 1 (LR swipe). As shown in Fig. 4(b), as the hand moves from left to right, the target signal of the left radar (R_L_) can be seen moving away from it. In contrast, the target signal can be seen moving towards R_R_. For the case of the radar used, a distance of 1 meter contains 156 fast times. Every sample consists of 90 slow time frames, which is equivalent to a duration of 4.5 seconds.

## Data Records

**Fig. 5:**
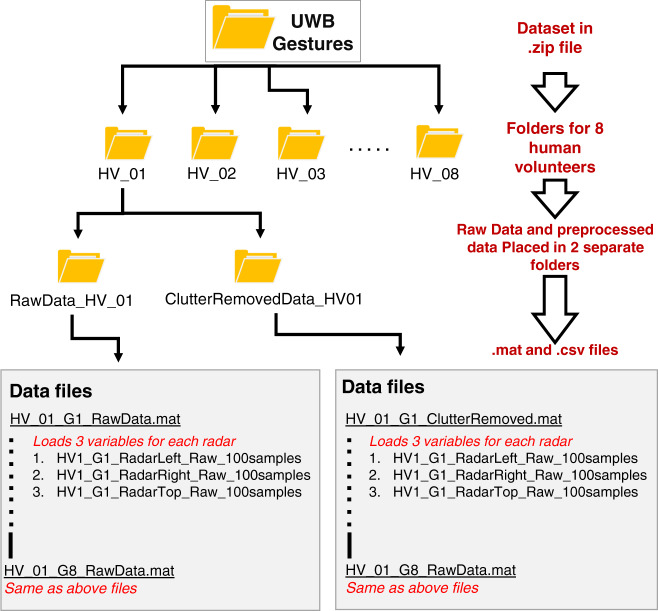
The structure of the UWB-Gestures dataset.

The UWB-Gestures dataset is available for download at Figshare^31^ (10.6084/m9.figshare.12652592). The data are placed in two separate folders to comply with the file size limit. Since the data were gathered using MATLAB, the stored files are MAT files. Additionally, to ensure license-free distribution of the dataset, we converted the dataset to a comma-separated values (CSV) file. For clarity, the modality video of each gesture is available on our homepage. The structure of the data descriptor is shown in Fig. 5. The dataset contains eight directories corresponding to each of the individual participants listed in Table 1. Each folder also contains two directories containing the raw data and clutter-removed data. The raw data comprise the recorded gestures in raw form, whereas the clutter-removed data consist of a preprocessed version of the raw data. During preprocessing, the clutter is estimated using the loopback filter^29^ based on the following principle:

3 $${c}_{n}\left[k\right]=\propto {c}_{n}\left[k-1\right]+(1-\propto ){x}_{n}[k]$$

where c represents the clutter term, which is extracted using the previously estimated clutter and the current received radar signal x[n], and the alpha (α) term represents the weighting factor that controls the learning rate of the filter. Particularly, for our dataset, alpha was chosen as 0.9. The estimated clutter c is then subtracted from the received radar signal x to obtain the clutter-free output y.

4 $${y}_{n}\left[k\right]={x}_{n}[k]-{c}_{n}[k]$$

As an example demonstrating how to access the subfiles in the folders, the link to access the files containing all the clutter-removed samples of gesture 4 (DU swipe) performed by human volunteer 2 is shown below:

~\UWB-Gestures\HV_02\ClutterRemovedData_HV02\HV_02_G4_ClutterRemoved.mat

Here, HV_02 refers to human volunteer 2, and G4 refers to gesture 4. The final MAT files containing separate variables corresponding to the three different radar systems are expressed as follows:

- **Left Radar:** HV2_G4_RadarLeft_ClutterRemoved_100samples.
- **Top Radar:** HV2_G4_RadarTop_ClutterRemoved_100samples.
- **Right Radar:** HV2_G4_RadarRight_ClutterRemoved_100samples.

Note that all the variables representing each radar value are saved as separate CSV files, resulting in three times more CSV files than MAT files. All the samples of each gesture are placed in a 2D file with the fast time on the horizontal axis and the slow time on the vertical axis. As stated above, every group of 90 slow-time values constitutes 1 gesture sample. A MATLAB script to access and view the hand gesture samples is also included and discussed in a later section in detail.

## Technical Validation

### Signal pattern analysis

**Fig. 6:**
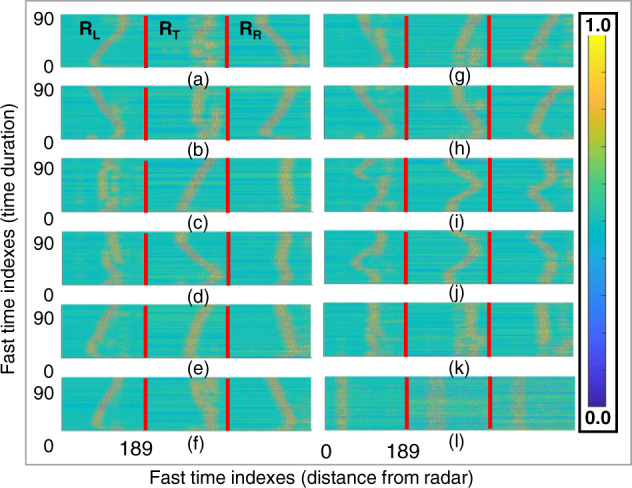
Data matrices for each radar sensor generated for all the performed gesture samples.

Figure 6 presents the signal patterns for all the remaining gestures. As seen in Fig. 6, each hand gesture movement corresponds to a distinctive pattern. As a practical example, for the LR swipe and RL swipe cases, the right and left radar sensors show opposite patterns, whereas radar 3 shows a straight vertical line. On the other hand, for the UD swipe and DU swipe cases, the left and right radars show a straight vertical line pattern, while radar 3 showed a varying pattern. Similar variations can be observed for each gesture. Similarly, Fig. 6(i) and (j) present the radar images corresponding to clockwise and counterclockwise rotational gestures, respectively.

### Example with a CNN-based classifier

**Fig. 7:**
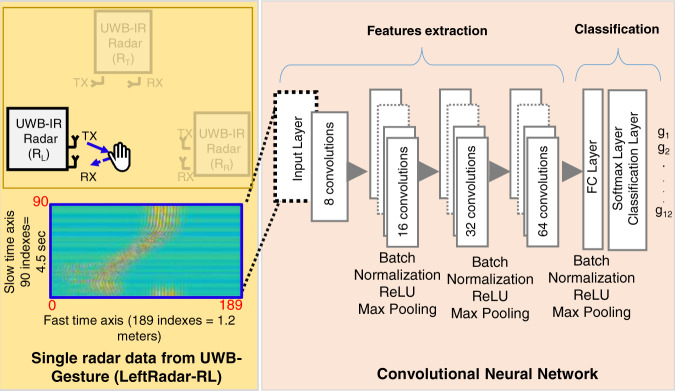
Example of the DCNN-based classifier for the proposed data descriptor.

To provide an example of applying the proposed dataset, we implement a novel DCNN-based classifier with four hidden layers, as shown in Fig. 7. The radar data matrix is converted into images, and these images are fed as input to the DCNN architecture. We perform classification using only a single radar sensor (left radar). Consequently, the size of the input layer to the DCNN is 90 × 189 (fast time × slow time of the single radar data matrix). We employ a 3 × 3 convolutional filter for each of the four hidden layers. The learning rate is set to 0.01, and 30 epochs are used for training purposes.

Table 3 presents the classification accuracy of the 4-layer DCNN algorithm shown in Fig. 7 with input from only the left radar sensor (R_L_). The first column of each row represents the original class, whereas the first row represents the predicted class of gestures. The diagonal values represent the overall success rate, and the values found elsewhere are the classification errors. In Table 3, the diagonal terms representing the success rate are marked in bold for clarity. The classifier based on the 4-layer DCNN architecture yielded an accuracy of 94% for the single radar sensor.

**Table 3:** Classification accuracy of the CNN-based classifier.

	a	b	c	D	e	f	g	h	i	j	k	l
**a**	**90.6**	0.0	0.0	0.0	0.0	0.0	0.7	0.0	0.0	0.7	0.0	1.4
**b**	0.0	**95.0**	2.0	0.0	0.0	0.0	0.0	0.0	0.0	0.7	0.0	0.0
**c**	0.0	0.0	**97.0**	0.0	0.0	0.0	0.0	0.0	0.0	0.0	0.0	0.0
**d**	0.0	0.0	1.0	**94.2**	0.0	0.0	0.0	1.4	0.0	0.0	0.0	0.0
**e**	0.0	0.0	0.0	3.6	**97.8**	0.0	0.0	0.0	0.0	0.7	2.9	0.0
**f**	1.0	0.7	0.0	0.0	0.0	**87.8**	5.0	0.7	0.0	0.0	0.0	0.7
**g**	2.0	4.3	0.0	0.0	0.0	11.5	**90.6**	0.7	2.8	0.7	2.2	0.0
**h**	0.0	0.0	0.0	2.2	0.0	0.0	1.4	91.4	3.2	0.0	0.0	0.0
**i**	0.0	0.0	0.0	0.0	0.0	0.7	0.0	5.8	**94.0**	0.0	0.0	0.7
**j**	0.0	0.0	0.0	0.0	0.0	0.0	0.0	0.0	0.0	**90.6**	1.4	0.0
**k**	2.2	0.0	0.0	0.0	2.2	0.0	2.2	0.0	0.0	6.5	**93.5**	4.3
**l**	4.2	0.0	0.0	0.0	0.0	0.0	0.0	0.0	0.0	0.0	0.0	**92.8**
**Overall classification accuracy for a single radar**												**94%**

## Data Availability

The files uploaded to Figshare^31^ also contain two independent programs for data visualization and for the example demonstration of the DCNN network presented in the above section. The first program can be used to generate the distance vs. time graph for user 1, as shown in Fig. 6. The dataset, its subfolders and the code (having the ‘m’ file extension) are extracted to the same directory where the code can be executed. After running the code, the user-interface instructions and comments in the code can be used to plot the distance-time (fast time vs. slow time) samples of the hand gestures. The same program can be used to plot the graphs for the other human volunteers as well. The second program is uploaded to a separate directory called “Exemplary CNN demonstration”, which can generate results similar to those shown in Table 3. Note that the exact accuracy may vary across trials.
